# 

**Published:** 1901-08-31

**Authors:** 


### NOTICE TO CORRESPONDENTS.

All MSS., Letters, Books for Review, and other matters intended for the Editor should be addressed The Editor, "The Hospital," The Hospital Building, 28 & 29 Southampton Street, Strand, W.C.

The Editor cannot undertake to return rejected MS., even when accompanied by stamped directed envelope.

**Notice to Advertisers and Subscribers.**

All Advertisements, Orders for Copies of The Hospital, and Business Communications should be addressed to **THE MANAGER (Not to the Editor),** The Hospital, 28 & 29 Southampton Street, Strand, London, W.C.

The Cost of Subscription, post free, is as follows:—Per week, 2½d.; per quarter, 2s. 8d.; per half-year, 5s. 3d.; per annum, 10s. 6d. Foreign:—Per annum, 16s. 2d., payable, in all cases, in advance.

**NOTICE.—Vol. XXIX. of "The Hospital" (October 1900, to March, 1901), in handsome cloth binding is now on sale at the Office of this Journal, price, 7s. 6d. Cases for binding the Numbers contained in this Volume 1s. 6d. Index to Vol. XXIX., 2½d., post free.**

**The Monthly Part for September will be ready on September 2. Price 6d., by post 8d.**

### Scraps and Gleanings.

The Children's Camp at Barton, near Winscombe, which has been built in memory of the Rev. U. R. Thomas, was recently opened. It has cost nearly £85.

The Cumberland and Westmorland section of the Co-operative Union have in contemplation a scheme providing for the establishment of a convalescent home for co-operators at an estimated cost of about £19,000.

In the report just issued, Dr. Boyd, the medical officer for South Shields, strongly denounces the overcrowded condition of the Denes Hospital at South Shields, on the scarlet fever side, where it has frequently happened that two patients have had to share one bed.

The first co-operative convalescent home to be established in this country was recently opened. This home has been built by the Co-operative Wholesale Society, and £10,000 was voted by the shareholders to carry out the scheme. It is situated near Shrewsbury.

The Women's Memorial to Queen Victoria for the county and city of Cork is to take the form of an endowment fund in aid of the Queen Victoria Jubilee Institute for Nurses. A subscription list has been opened, and county and city committees have been formed.

The Hospital Saturday collections at Blackpool this year show a falling off as compared with last year. This decrease is attributed to the fact that only 11 ladies undertook the work of collecting this year, whereas last year there were 40 engaged on the task, and they collected £177.

The county of Worcester has in hand a scheme for the establishment in the county of sanatoria for the treatment of consumption. The plans drawn up provide accommodation for 48 patients, and the site for the sanatorium has already been placed at the committee's disposal by Viscount Cobham. It is 30 acres in extent, and is practically a pine wood.

The Committee of the Hospital of St. Francis, New Kent Road, are endeavouring to raise sufficient funds to enable them to purchase the house adjoining, with a view to extending the present buildings. A sum of £750 is required to complete the necessary amount, and Lord Llangattock has promised the last £100, whilst Mr. A. F. Hills has promised the last £100 but one.

"A Civilian War Hospital," published by John Murray, is the complete record of the Portland Field Hospital, which was the first civilian hospital to be equipped and sent out to the front after the declaration of war. The establishment and equipment, etc., of this hospital was the work of Mrs. Bagot, who was generously assisted with funds by the Duke of Portland and others. The records in question are by the medical staff.

The Oldham Jubilee committee have in hand a sum of £13,134 for the purpose of establishing the scheme of commemoration of Queen Victoria's reign, which is to take the form of substantially increasing the accommodation for in-patients at the Oldham Infirmary. The secretary to the committee announced at their recent meeting that since February they had received a sum of £1,000 from the workpeople and £1,000 from Messrs. Platt. A total sum of £18,000 is required to carry out the work according to the amended plans which have been approved, and which provide for 53 beds, so that the committee have still to collect £5,000.

### The Student of Medicine.

### APPOINTMENTS.

J. H. Battersby, M.D., C.M.Edin., appointed Medical Officer of the Doncaster Workhouse. **H. R. Beale,** M.R.C.S., L.R.C.P., appointed Assistant Resident Medical Officer to the Leeds General Infirmary. **G. A. Carter,** M.R.C.S., L.R.C.P.Lond., appointed Casualty Officer to the Hull Royal Infirmary. **Harold Coates,** M.D., D.P.H. (Vict.), appointed Medical Officer of Health of the Borough of Burton-upon-Trent. **F. Collins,** M.R.C.S., L.R.C.P.Lond., appointed Medical Officer of Health of the Sawbridgeworth Urban District. **Rowland W. Collum,** M.R.C.S., L.R.C.P., appointed Second Anæsthetist to the Hospital for Sick Children, Great Ormond Street, W.C. **Charles H. Fennell,** M.A., M.B., B.Ch.Oxon., M.R.C.S., L.R.C.P., appointed Medical Registrar to the Hospital for Sick Children, Great Ormond Street, W.C. **V. Graham,** M.R.C.S., L.R.C.P.Lond., appointed Medical Officer of the East District of the Doncaster Union. **O. Gruner,** M.R.C.S., L.R.C.P., appointed House Physician to the Leeds General Infirmary. **W. Hanna,** M.B., B.Ch., B.A.O., R.U.I., D.P.H.Camb., appointed Assistant Medical Officer of the Port of Liverpool. **E. Hnnt,** M.R.C.S., L.R.C.P.Lond., appointed District Medical Officer of the Newton Abbot Union. **William Hunter,**

M.B., C.M.Aberd., appointed Assistant Director of the Pathological Institute, London Hospital, E. **P. McDonald**, M.B., C.M.Aberd., appointed District Medical Officer of the Great Ouseburn Union. **E. Thurlow Potts**, appointed Resident Medical Officer to the Hospital for Sick Children, Newcastle-on-Tyne. **Nathan Raw**, M.D., M.R.C.P.Lond., F.R.S.E., D.P.H., appointed Visiting Medical Superintendent to the West Derby Union Infirmary, Liverpool. **C. Lawson Smith**, M.B., Ch.B.Aberd., appointed Senior Resident Medical Officer to the Tottenham Hospital, N. **W. Strover**, M.R.C.S.Eng., L.S.A., appointed Medical Officer of Health of the Chingford Urban District. **A. M. Watts**, M.R.C.S., L.R.C.P.Lond., appointed Medical Officer of Health for East Elloe Rural District. **A. J. Young**, L.R.C.P., and S.Edin., L.F.P.S.Glasg., appointed Medical Officer of Health of the Whitefield Urban District.

